# Pigment Epithelium-Derived Factor Promotes Axon Regeneration and Functional Recovery After Spinal Cord Injury

**DOI:** 10.1007/s12035-019-1614-2

**Published:** 2019-05-02

**Authors:** Andrew R. Stevens, Umar Ahmed, Vasanthy Vigneswara, Zubair Ahmed

**Affiliations:** 1 Neuroscience and Ophthalmology, Institute of Inflammation and Ageing, College of Medical and Dental Sciences, Robert Aitken Institute of Clinical Research, University of Birmingham, Birmingham, B15 2TT UK; 2 King Edward VI Camp Hill School for Boys, Vicarage Road, Kings Heath, Birmingham, B14 7QJ UK

**Keywords:** PEDF, Spinal cord injury, Serum withdrawal, Dorsal root ganglia neurons, CNS, Axon regeneration, Neurite outgrowth

## Abstract

Although neurons in the adult mammalian CNS are inherently incapable of regeneration after injury, we previously showed that exogenous delivery of pigment epithelium-derived factor (PEDF), a 50-kDa neurotrophic factor (NTF), promoted adult retinal ganglion cell neuroprotection and axon regeneration. Here, we show that PEDF and other elements of the PEDF pathway are highly upregulated in dorsal root ganglion neurons (DRGN) from regenerating dorsal column (DC) injury paradigms when compared with non-regenerating DC injury models. Exogenous PEDF was neuroprotective to adult DRGN and disinhibited neurite outgrowth, whilst overexpression of PEDF after DC injury in vivo promoted significant DC axon regeneration with enhanced electrophysiological, sensory, and locomotor function. Our findings reveal that PEDF is a novel NTF for adult DRGN and may represent a therapeutically useful factor to promote functional recovery after spinal cord injury.

**Electronic supplementary material:**

The online version of this article (10.1007/s12035-019-1614-2) contains supplementary material, which is available to authorized users.

## Introduction

Injury to the central nervous system (CNS) is a considerable cause of morbidity and mortality in modern populations. The intrinsic inability of the CNS to repair on a cellular level leads to poor outcomes for rehabilitation on a patient level. Neuronal cells lost through injury are not replaced, and axons do not regenerate; the damage acquired causes a permanent functional deficit. Whilst spinal cord injury (SCI) presents a pertinent clinical problem, no regenerative therapy is available which offers an adequate level of functional recovery [1]. Pigment epithelium-derived factor (PEDF) is a 50-kDa glycoprotein and a member of the serine protease inhibitor (serpin) gene family, with neuroprotective and angiogenic properties. PEDF has been noted for its neuroprotective activity throughout the CNS [2, 3]. Whilst PEDF has been shown to promote survival and neurite outgrowth of motor neurons, evidence is limited to in vitro studies utilising embryonic avian cultures [4]. More recently PEDF has been shown to be both neuroprotective and axogenic in adult retinal ganglion cells after optic nerve crush injury [5, 6]. We therefore asked the question as to whether PEDF is neuroprotective and axogenic to spinal neurons.

To test this hypothesis, we utilised dorsal root ganglion neurons (DRGN), whose cell bodies subserve both the central and peripheral projections, yet these neurons can regenerate their peripheral projections but not their central projections. These DRGN can be placed under different in vivo conditions to create regenerating and non-regenerating models of CNS injury [7–11]. A non-regenerating SCI can be modelled by administering a dorsal column (DC) crush injury; DRGN axons barely regenerate but do not extend into the DC injury site. A peripheral regenerating paradigm can be modelled by a sciatic nerve (SN) crush injury; axons will extend into the SN injury site. By combining these models, pre-conditioning (p) SN lesions, i.e. administering an SN crush lesion 7 days prior to DC injury (pSN + DC), the central axons will extend into and through the DC injury site as well as peripheral axons extending into the SN lesion site [8]. Pre-conditioning is explained by upregulation of gene transcription, which enables DRGN to enter a ‘growth-activated state’, rendering them capable of regenerating their axons into the DC lesion site [10–13].

Here, we investigated DRGN survival and regeneration in response to PEDF in the adult spinal cord, evaluating the potential for PEDF to be of value as a regenerative therapy in the clinical setting. We show that PEDF is activated in regenerating models of DC injury, promotes DRGN survival and neurite outgrowth in a serum-withdrawal model in vitro, promotes DC axon regeneration and improved functional recovery in vivo, and causes the release of several Trk-dependent neurotrophic factors (NTF) that account for some of its survival/neurite-growth promoting effects.

## Methods

### Regenerating and Non-Regenerating DC Injury Models

All surgical procedures were licenced by the UK Home Office and ethically approved by the University of Birmingham’s Animal and Ethical Review Board. Surgeries were carried out in strict accordance to the guidelines of the UK Animals Scientific Procedures Act, 1986, and the Revised European Directive 1010/63/EU and conformed to the guidelines and recommendation of the use of animals by the Federation of the European Laboratory Animal Science Associations. Adult female Sprague-Dawley rats (170–220 g) (Charles River, Margate, UK) were randomly allocated to either: (1) Sham (control; partial laminectomy but no DC crush injury); (2) DC crush injury (non-regenerating; partial laminectomy followed by DC crush injury); (3) SN crush injury (regenerating SN); and (4) pre-conditioning (p) SN crush injury (pSN) 1 week prior to DC crush injury (pSN + DC). Where specified intact animals with no surgical procedures were also used. All lesions were administered under 5% isoflurane-induced inhalation anaesthesia, with 1.5 l/min O_2_. Pre- and post-injury analgesia was also provided.

DC crush injury was administered bilaterally at the T8 vertebral level as described by us previously [9, 14]. Briefly, calibrated watchmaker’s forceps were inserted through the dorsal cord meninges to a depth of 1.5 mm, and a DC crush was performed. SN crush was performed on the left SN, at the level of the sacro-tuberous ligament, exposed at mid-thigh level. DC injury with pre-conditioning SN injury models were carried out as described, with SN injury preceding DC injury by 1 week.

All in vivo experiments were performed by investigators masked to the treatment conditions with animals randomly assigned to treatment groups. For the microarray experiments, four independent samples of DRG were from two pooled DRG per experimental group from intact, DC, SN, and pSN + DC animals to ensure enough high-quality RNA could be extracted (*n* = 8 rats/group, total for experiment = 32 rats). For quantitative RT-PCR (qRT-PCR), RNA from four DRG/group (*n* = 4 rats/group) were extracted to validate highly changed genes in DC, SN, and pSN + DC animals and repeated on three independent occasions (*n* = 12 rats/group, total for experiment = 36 rats). For immunohistochemistry to determine PEDF and PEDF-R localisation, three DRG/group were used and repeated on three independent occasions (*n* = 9 rats/group, total for experiment = 36 rats). To determine levels of PEDF by Western blot in L4/5 DRG from intact and after DC, SN and pSN + DC injuries, total protein extracts were prepared from *n* = 3 rats/group and repeated on three independent occasions (*n* = 9 rats/group, total for experiment = 36 rats).

To determine if PEDF overexpression by viral vector-mediated (adeno-associated vector, serotype 8—AAV8) delivery to DRGN promotes DC axon regeneration and functional recovery (sensory and locomotor), *n* = 6 rats/group were used, repeated on three independent occasions, comprising: Sham, DC + AAV-Null (control) and DC + AAV-PEDF (total for experiment = 54 rats). Rat PEDF was overexpressed in DRGN using AAV8 under the control of a CMV promoter, referred to as AAV-PEDF from herein (AAV8-rat-SERPINF1; cat no. AAV-291714; Vector Biolabs, Malvern, PA, USA). An AAV8-Null vector under the control of a CMV promoter, referred to as AAV-Null from herein (AAV8-Null; Cat no. 7077, Vector Biolabs) was used to control for AAV-PEDF effects in vivo. 1 × 10^13^ viral particles of each vector in a final volume of 5 μl of PBS was injected directly into the DRG (intra-DRG) as described by us previously [15]. Axon regeneration and sensory and locomotor function were assessed at 6 weeks after SCI and treatment, as described later. Electrophysiology (*n* = 6 rats/group, 3 independent repeats, total for experiment = 54 rats) was also performed at 6 weeks after injury and treatment, as described below.

Since AAV8-mediated overexpression is not translational as it requires 1–2 weeks to reach maximum expression of the transgene, we used in vivo-jetPEI, a non-viral vector, which is just as efficient as AAV8, transducing up to 30% of DRGN after intra-DRG injection [16]. Therefore, we determined if PEDF overexpression by in vivo-jetPEI (referred to as PEI from herein; Polyplus Transfection, NY, USA) promoted functional recovery after DC injury. We used *n* = 6 rats/groups comprising: Sham, DC + Vehicle, DC + PEI-Null, and DC+PEI-PEDF, repeated on three independent occasions (total for experiment = 72 rats) to perform electrophysiology, tape sensing, and removal and ladder crossing tests as described below. DC crush, preparation and injection of PEI into L4/L5 DRG, electrophysiology, and functional tests were performed as described by us previously [16]. Rats were killed at either 28 days for qRT-PCR and enzyme-linked immunosorbent assay (ELISA) to determine levels of PEDF overexpression or at 6 weeks to perform electrophysiology [16].

All other DRGN culture experiments used a total of 60 rats (4 rats/group, run in duplicate and repeated on 3 independent occasions). Animals were killed by exposure to rising concentrations of CO_2_.

### Microarray Analysis

Groups of four rats (randomised and identity masked) comprising intact control, DC, SN, and pSN + DC injured animals were treated as described above, and L4/L5 DRG pairs were harvested from each animal and RNA extracted was extracted using TRIzol reagent following the manufacturer’s protocol (Invitrogen, Paisley, UK). The quality of extracted RNA was checked using a Bioanalyzer. The rat genome AROS™ V3.0set (Operon Biotechnologies GmbH, Cologne, Germany) containing 26,962 long-mer probes representing 22,012 genes and 27,044 gene transcripts was used for the microarray analysis as described by us previously [17].

### RNA Extraction and Quantitative RT-PCR

Total RNA was extracted from harvested DRG (randomised and identity masked) at appropriate timepoints after injury with or without treatment using TRIzol reagent following the manufacturer’s protocol (Invitrogen). For cell culture experiments, total RNA was extracted directly from wells following the manufacturer’s instructions. The levels of mRNA of selected genes were determined using pre-validated rat primer sequences from complimentary DNA prepared from extracted mRNA, and qRT-PCR was performed using a LightCycler PCR machine (Roche, Burgess Hill, UK) [18]. Primer sequences included PEDF-Rn00709999_m1; Akt-Rn00583646_m1; p50-Rn00550870_m1; SRF-Rn01757240_m1; BDNF-Rn02537967_S1; NGF-Rn01533872_m1; GDNF-Rn00569510_m1; Bcl2-Rn99999125_m1 (all from Thermo Fisher Scientific, Leicestershire, UK). Fold changes were computed using the ΔΔCt method [18].

### Pathway Analysis

Data from mRNA microarray studies of DRGN models of injury was utilised to analyse all genes in the PEDF pathway. Gene expression for each model was standardised as fold increase compared with intact DRGN. Genes significantly altered (greater or lower than 2-fold) in injury models were represented in a schematic pathway with their corresponding fold changes.

### Immunohistochemistry

After killing animals by exposure to rising concentrations of CO_2_, rats (randomised and masked) were intracardially perfused with 4% formaldehyde (TAAB Laboratories, Berkshire, UK) in 0.1 M phosphate-buffered saline (PBS). DRG were removed from vertebral levels L4 and L5, post-fixed in 4% formaldehyde and subjected to a graded series of sucrose solutions for cryoprotection. Tissues were blocked in OCT mounting compound (TAAB laboratories) and sectioned at 15 μm-thick using a cryostat (Brights Instruments, Huntingdon, UK) before being adhered to charged glass slides (Thermo Fisher Scientific, Loughborough, UK). Slides were kept at − 20 °C until required. Immunohistochemistry was performed as described by us previously [14]. Briefly, slides were washed in three changes of PBS, followed by three further washes in PBS containing 1% (*v*/*v*) Triton X-100 (Sigma) (PBS-T) to permeabilise cells. Sections were then blocked for 1 h at room temperature (RT) using PBS containing 0.05% (*w*/*v*) bovine serum albumin (Sigma, Poole, UK) and 0.05% Tween-20 (Sigma) and incubated overnight (16–18 h) at 4 °C in a humidified chamber with appropriate primary antibodies: goat anti-PEDF (1:400 dilution, R&D Systems, Oxford, UK)); mouse anti-NF200 (1:400 dilution, Sigma); and rabbit anti-PEDF-R (1:500 dilution, R&D Systems). Slides were washed 3× in PBS-T before incubation for 1 h at RT with the relevant secondary antibody: Alexa488 anti-goat; Texas Red anti-mouse; Alexa594 anti-rabbit, all diluted at 1:400, all from Invitrogen). Slides were washed twice in PBS-T, followed by twice in PBS. Coverslips were then mounted using Vectashield mounting medium (containing DAPI) (Vector Laboratories, Peterborough, UK). Negative controls were included in each run where primary antibodies were omitted, and these slides were used to set the background threshold levels prior to image capture.

### Protein Extraction, Western Blot, and Densitometry

Total protein, Western blot, and subsequent densitometry were performed as described by us previously [5]. Briefly, total protein was extracted from L4/L5 DRG from three rats (randomised and masked; repeated on 3 independent occasions, total *n* = 9 rats/group) after DC injury and pooled together to ensure enough protein. Forty micrograms of total protein was resolved on 12% SDS polyacrylamide gels and blotted onto polyvinylidene fluoride (PVDF) membranes (Millipore, Watford, UK) and probed with relevant primary antibodies: goat anti-PEDF (1:500 dilution, R&D Systems) and β-actin (1:1000 dilution, Sigma; protein loading control). Membranes were then incubated with relevant HRP-labelled anti-goat and anti-mouse IgG secondary antibodies and bands were detected using the enhanced chemiluminescence kit (GE Healthcare, Buckinghamshire, UK).

For densitometry, Western blots were scanned into Adobe Photoshop (Adobe Systems Inc., San Jose, CA, USA), and the integrated density of bands was analysed using the built-in-macros for gel analysis in ImageJ (NIH, USA, http://imagej.nih.gov/ij) by an investigator masked to the treatment conditions [5, 11, 19]. Means ± SEM were plotted in Microsoft Excel (Microsoft Corporation, CA, USA).

### Neurotrophic Factor Enzyme-Linked Immunosorbent Assay

The levels of brain-derived neurotrophic factor (BDNF), glial-derived neurotrophic factor (GDNF), and nerve growth factor (NGF) in culture were detected by ELISA using the appropriate rat ELISA kits, according to the manufacturer’s instructions (Abcam, Cambridge, UK) by an investigator masked to the treatment conditions. A rat PEDF ELISA kit was used to detect PEDF in DRG after intra-DRG injection of AAV-PEDF and after pSN + DC lesions, according to the manufacturer’s instruction (Elbascience, Houston, TX, USA). ELISA was performed on 10 μl of each sample, in duplicate, and repeated on three independent occasions.

### Adult DRGN Primary Cell Cultures

For primary DRGN cultures, adult (170–220 g) female Sprague-Dawley rats (Charles River) were used as described by us previously [20]. Briefly, DRG pairs (T1-L7) were removed and dissociated into single cells using 0.1% collagenase (Sigma). DRGN were then centrifuged through a 15% BSA gradient and the resultant cell pellet was re-suspended in Dulbecco’s modified eagle medium (DMEM) in 8-well glass chamber slides (BD Biosciences, Oxford, UK), pre-coated with 100 μg/ml poly-d-lysine and 20 μg/ml laminin (both from Sigma). Dissociated DRGN were plated at 500 cells/well with (DMEM) containing 1% penicillin-streptomycin (PS) (all from Invitrogen) and 30 μM 5-fluoro-2-deoxyuridine (5-FDU, Sigma) to limit non-neuronal cell proliferation [20].

### In Vitro Serum Withdrawal Model

For the serum withdrawal model, DRGN were plated in 500 μl of DMEM/foetal bovine serum/penicillin-streptomycin in 8-well chamber slides, as described by us previously [5]. After 20–24-h incubation, the medium was replaced with DMEM: with or without serum, and with or without human recombinant PEDF (Peprotech, London, UK) in the presence of 5-FDU. K252a was used at a final concentration of 50 nM whilst Trk/A/B/C-Fc fusion proteins were used at 5 μg/ml each [21]. Chamber slides were incubated for 120 h before being fixed in 4% formaldehyde and subsequent immunocytochemistry, as described below. All experiments were performed in duplicate and repeated on four independent occasions (*n* = 8 wells/condition) by an investigator masked to the treatment conditions.

### Treatment of DRGN and ELISA to Determine NTF Concentrations

DRGN were treated in duplicates (*n* = 8 wells/condition) with or without PEDF, culture media was collected after 120 h, and samples were assayed to determine the concentrations of NTF using rat-specific ELISA kits, according to the manufacturer’s protocol (R&D Systems) by an investigator masked to the treatment conditions.

### Immunocytochemistry

Immunocytochemistry was performed as described by us previously [5] by an investigator masked to the treatment conditions. Briefly, cultures were fixed for 10 min using 4% formaldehyde, followed by ×3 washes in PBS. Non-specific staining was blocked for 10 min in PBS containing 3% BSA (*w*/*v*) and 1% Triton X-100, followed by incubation with primary antibody: mouse anti-βIII-tubulin (to mark DRGN soma and neurites, diluted 1:200) for 1 h at RT, diluted in PBS with 3% BSA and 0.5% (*v*/*v*) Tween-20. Plates were washed and incubated with Alexa488-labelled anti-mouse IgG secondary antibody (Invitrogen, 1:400 dilution) for 1 h before mounting in Vectamount with DAPI (Vector Laboratories).

### DRGN Survival and Neurite Outgrowth

With the experimenter masked to the treatment conditions, mean numbers of βIII-tubulin^+^ DRGN was counted in 9 quadrants/well and the total number of DRGN determined as described before [20]. The number of DRGN with neurites and the mean neurite lengths were also quantified in 9 quadrants/well. The longest neurite was measured using Axiovision (Zeiss) from at least 180 DRGN/condition, whilst total DRGN counts to assess survival were made in all wells.

### Microscopy and Image Analysis

Immunostained sections were viewed under an epi-fluorescent microscope (Axioplan 200) equipped with an Axiocam HRc and Axiovision software (all from Zeiss, Hertfordshire, UK). For each experiment, exposure times were set to optimise images of control slides and adjusted to account for background staining in negative controls (no primary antibody). An investigator masked to the treatment conditions performed image capture and analysis.

To quantify fluorescence, images were captured at × 10 magnification of the entire DRG section, each taken from the same depth through the DRG, adjusted equally in Photoshop, and merged to a composite image using the Photomerge tool. In ImageJ (National Institutes of Health, USA), six areas of DRGN/section (*n* = 12 DRG/condition) were selected and mean pixel counts recorded.

To measure neurite outgrowth, images of DRGN immunostained for βIII-tubulin were captured for 30 randomly selected DRGN per well. Using Axiovision Software (Carl Zeiss), the number of DRGN with neurites and the length of the longest neurite were recorded from a minimum of 180 DRGN/condition [20]. Image capture and subsequent analyses were performed by an investigator masked to the treatment conditions.

### Quantification of Axons

Axon regeneration in the spinal cord was quantified using GAP43^+^ immunoreactivity, according to previously published methods [22] by an investigator masked to the treatment conditions. GAP43 was used since Cholera toxin B labelling did not work in the rat [22]. Briefly, serial parasagittal sections of cords were reconstructed by collecting all serial 50-μm-thick sections (∼ 70–80 sections/animal; *n* = 18 rats/treatment), and the numbers of intersections of GAP43^+^ fibres through a dorsoventral-orientated line were counted by a masked investigator, from 4 mm rostral to 4 mm caudal to the lesion site. Finally, axon number was calculated as a percentage of the fibres seen 4 mm above the lesion, where the DC was intact by an investigator masked to the treatment conditions.

### Electrophysiology

Compound action potentials (CAP) were recorded at 6 weeks after DC injury and treatment, as described previously [16]. Briefly, the experimenter was masked to the treatment status of the animals and the CAP amplitude was calculated between the negative deflection after the stimulus artefact and the next peak of the wave. CAP area was also calculated by rectifying the CAP component (full-wave rectification) and measuring its area. To confirm our recordings and that a CAP could not be recorded, the dorsal half of the spinal cord was transected after each experiment between the stimulating and recording electrodes.

### Functional Tests

Functional testing after DC lesion and treatment was carried out as described previously [16, 23]. Briefly, animals (*n* = 18/group) were first trained to master traversing a rope and a horizontal ladder for 1 week before functional testing. Baseline parameters were established by performing tests at 2–3 days before injury. Animals were then tested at 2 days, 1 week, 2 weeks, 3 weeks, 4 weeks, 5 weeks, and 6 weeks after DC injury + treatment. Experiments were performed by an observer masked to the treatment conditions in the same order and time of day with each test performed for three individual trials.

#### Horizontal Ladder Test

This tests the animal’s locomotor function and is performed on a 0.9-m-long horizontal ladder with a diameter of 15.5 cm and randomly adjusted rungs with variable gaps of 3.5–5.0 cm. Animals were assessed traversing the ladder, and the left and right rear paw slips were recorded along with the total number of steps and the mean error rate as: the number of slips/total number of steps.

#### Tape Removal Test (Sensory Function)

The tape removal test determines touch perception from the left hind paw. After holding animals with both hind paws extended, the time it took for the animal to detect and remove a piece of tape of 15 × 15 mm (Kip Hochkrepp, Bocholt, Germany) affixed to the palm of the left hind paw was recorded and used to calculate the mean sensing time.

### Statistical Analysis

Statistical significance was calculated from sample means using one-way analysis of variance (ANOVA) with post hoc Dunnett’s method using SPSS Statistics 19 (IBM, NY, USA).

For the horizontal ladder crossing and tape removal tests, data was analysed as described previously [16, 23] using R package (www.r-project.org). Briefly, for the ladder crossing test, whole time course of lesioned and Sham-treated animals were compared using binomial generalised linear mixed models (GLMM). Binomial GLMMs were fitted in R using package *lme4* with the *glmer* function. *P* values were then calculated using parametric bootstrap. For the tape removal test, linear mixed models (LMM) were calculated by model comparison in R using the package *pbkrtest*, with the Kenward-Roger method [16, 23].

## Results

### PEDF Signalling Pathway Is Upregulated in Regenerating Models of DC Injury

**Fig. 1:**
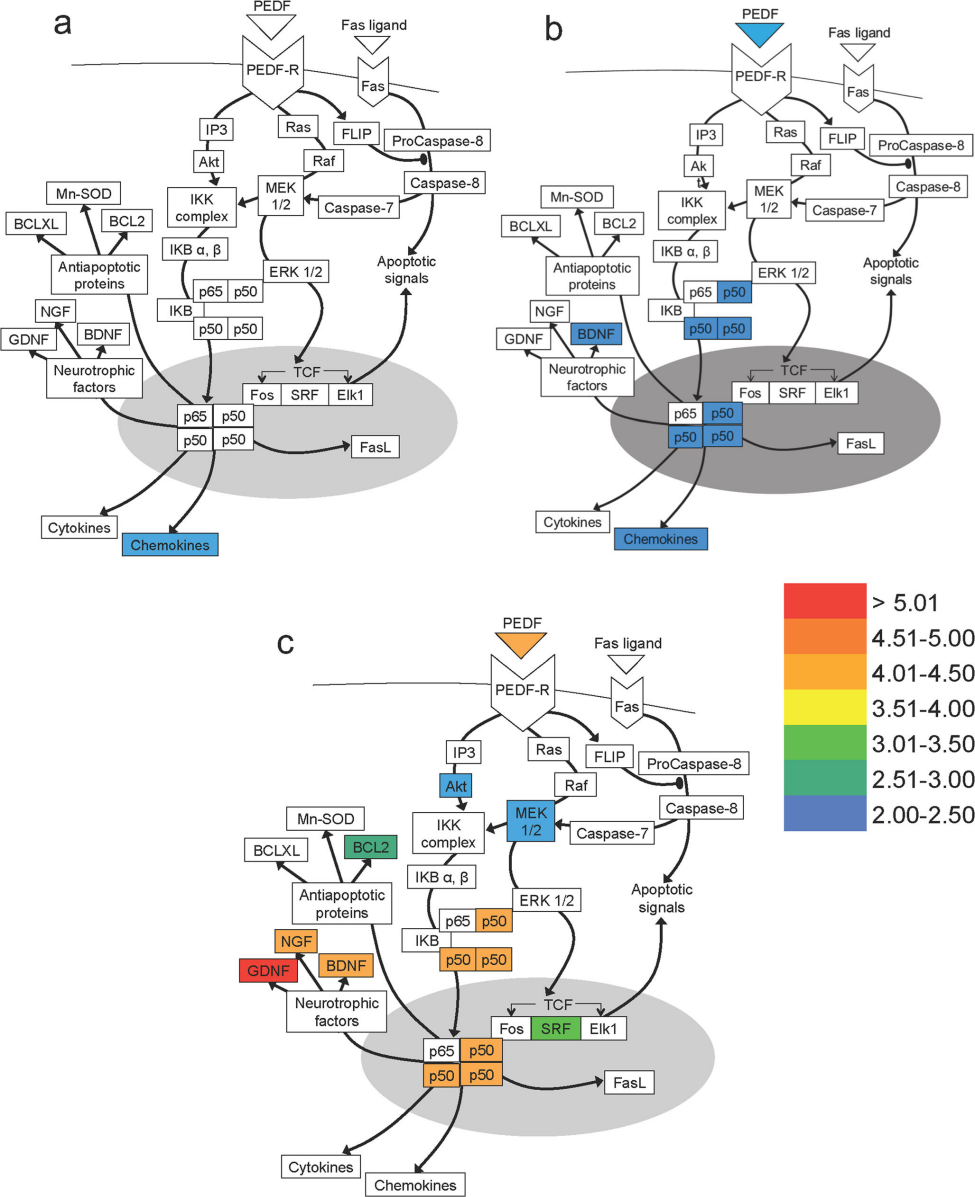
Microarray data showing changes in mRNA of molecules of the PEDF pathway in non-regenerating and regenerating DRGN models. Fold difference in mRNA extracted from DRG 10 days post-lesion after **a** DC, **b** SN, and **c** pSN + DC models. Fold increase in mRNA was normalised to the intact controls. Curved line indicates cell membrane. Grey oval indicates nucleus. Arrows indicate promoting interaction. Arrows with oval tips indicate inhibitory interactions. Colour chart indicates fold-increase by colour heat with indicated levels of gene expression (fold-changes compared with the intact controls)

To investigate endogenous genetic changes in non-regenerating DC and regenerating SN and pSN + DC, microarray analysis of mRNA levels in DRGN was performed at 10dpl. Out of the 156 genes currently known to be involved in the PEDF pathway, 53, 49, and 53 molecules in DC, SN, and pSN + DC models, respectively, showed any mRNA changes compared with the intact controls with all molecules showing significant changes (i.e. > 2-fold regulation) depicted in Fig. 1. In non-regenerating DC models, only 3 molecules in the PEDF pathway were upregulated > 2-fold: mRNA for chemokines CCL2, CCL5, and CCL6 were upregulated 2.22-, 2.55-, 2.01-fold, respectively (Fig. 1a). In regenerating SN models, 8 molecules changed > 2-fold, including the transcription factor NFκB (p50, p65) which were upregulated between 2.00- and 2.50-fold and BDNF was also upregulated by 2.27-fold (Fig. 1b). The most marked upregulation occurred in the regenerating pSN + DC model where 20 molecules changed > 2-fold (Fig. 1c). These included mRNA for PEDF, which was 4.33-fold upregulated and transcription factor NFκB (p50, p65) that was upregulated between 4.00- and 4.50-fold, respectively. Furthermore, there was a 2.55-fold upregulation of mRNA for anti-apoptotic protein Bcl2. In addition, three NTF were highly upregulated, including glial-derived neurotrophic factor (GDNF; 5.04-fold), nerve growth factor (NGF; 4.32-fold) and brain-derived neurotrophic factor (BDNF; 4.25-fold) (Fig. 1c). These results suggest that PEDF and other pathway molecules were positively correlated with DC axon regeneration.

### PEDF mRNA and Protein Are Present in High Levels in Regenerating SN and pSN + DC Paradigms

**Fig. 2:**
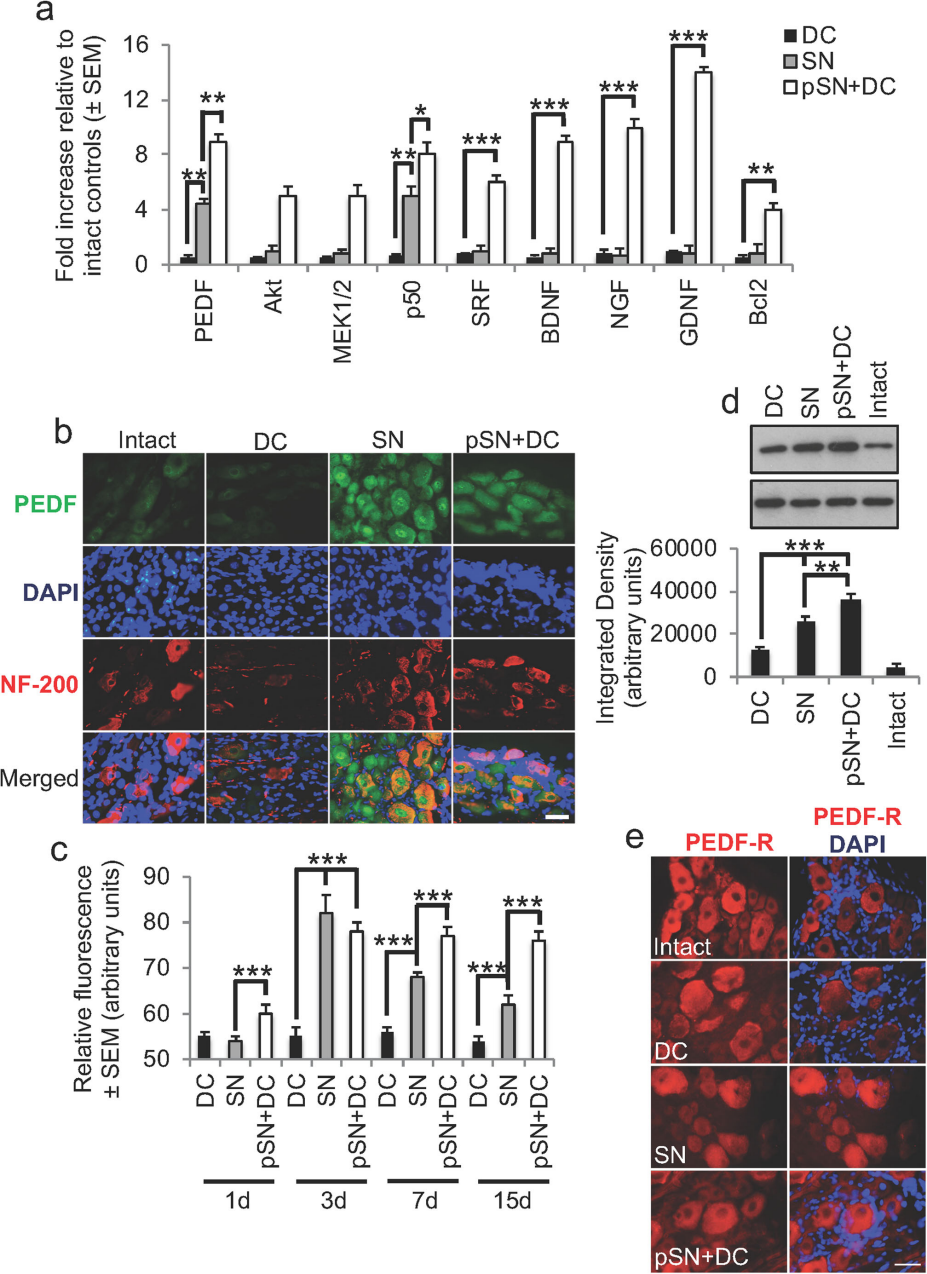
Confirmation of highly changed genes in pSN + DC DRGN by qRT-PCR and confirmation of changes in PEDF. **a** Confirmation of highly upregulated levels of PEDF, Akt, MEK1/2, p50, SRF, BDNF, NGF, GDNF, and Bcl2 by qRT-PCR. **b** Immunohistochemistry at 10 days after DC injury and **c** quantification of immunopositive staining at 1, 3, 7, and 15 days post-DC injury showed high levels of PEDF immunoreactivity (green) in regenerating SN and pSN + DC DRGN soma (red) (DAPI = blue, cell nuclei), whilst low levels were detected in intact and DC-injured DRGN. **d** Western blot and subsequent densitometry detected high levels of PEDF in regenerating SN and pSN + DC models, with the highest levels of PEDF being present in pSN + DC DRG. **e** PEDF-R (green) was present in DRGN and remained unchanged in intact and all injury models. DAPI (blue) = cell nuclei. Scale bars in **b** and **e** = 25 μm*.* * = *P*<0.05, ** = *P* < 0.001, ANOVA; *** = *P* < 0.0001, ANOVA

To validate the microarray changes observed, we performed qRT-PCR for a selection of the most highly changed genes and showed that the data corroborated our microarray findings. These results showed significant changes in expression of PEDF, Akt, MEK1/2, p50, SRF, BDNF, NGF, GDNF, and Bcl2 in pSN + DC models compared with SN and DC models (Fig. 2a). To investigate the relationship between PEDF and an enhanced growth-activated state of CNS neurons in vivo, PEDF and its relative levels were assessed in non-regenerating DC and regenerating SN and pSN + DC models of injury. PEDF immunostaining (green) was weakly positive in DRGN (red) in the intact control and non-regenerating DC models (Fig. 2b). However, PEDF immunostaining was highly positive in DRGN in regenerating SN and pSN + DC models (Fig. 2b). Interestingly, PEDF immunoreactivity in DRGN from regenerating SN models was localised to the nucleus (arrows) and DRGN somata, whilst in the regenerating pSN + DC model, immunoreactivity was mainly localised in DRGN somata.

Quantification of the immunoreactivity for PEDF over time after injury in non-regenerating and regenerating models showed that immunoreactivity reduced over time in regenerating SN models but remained significantly elevated (*P* < 0.0001, ANOVA for DC versus SN and pSN + DC at 3 days and *P* < 0.001 for SN versus pSN + DC at 7 days and *P* < 0.0001 for SN versus pSN + DC at 15 days) in regenerating pSN + DC models, compared with the DC and intact controls, for a period of at least 15 days after injury (Fig. 2c). Western blot and subsequent densitometry confirmed immunohistological changes demonstrating that SN and pSN + DC models contained the highest levels of PEDF, with significantly elevated levels (*P* < 0.001, ANOVA) present in pSN + DC models (Fig. 2d).

For DRGN to be able to respond to changes in exogenous PEDF, the PEDF-receptor (PEDF-R) must be present in DRGN. Immunostaining for PEDF-R (red) in DRGN was present in DRGN somata in the intact controls and remained unchanged in DC, SN, or pSN + DC models (Fig. 2e). Taken together, these results suggest that PEDF is upregulated in regenerating SN and pSN + DC injury paradigms and remains elevated throughout the first 15 days after injury, albeit more so in pSN + DC models. The PEDF-R is also present in DRGN and so are able to respond to changes in PEDF levels.

### PEDF Promotes DRGN Survival in a Dose-Dependent Manner

**Fig. 3:**
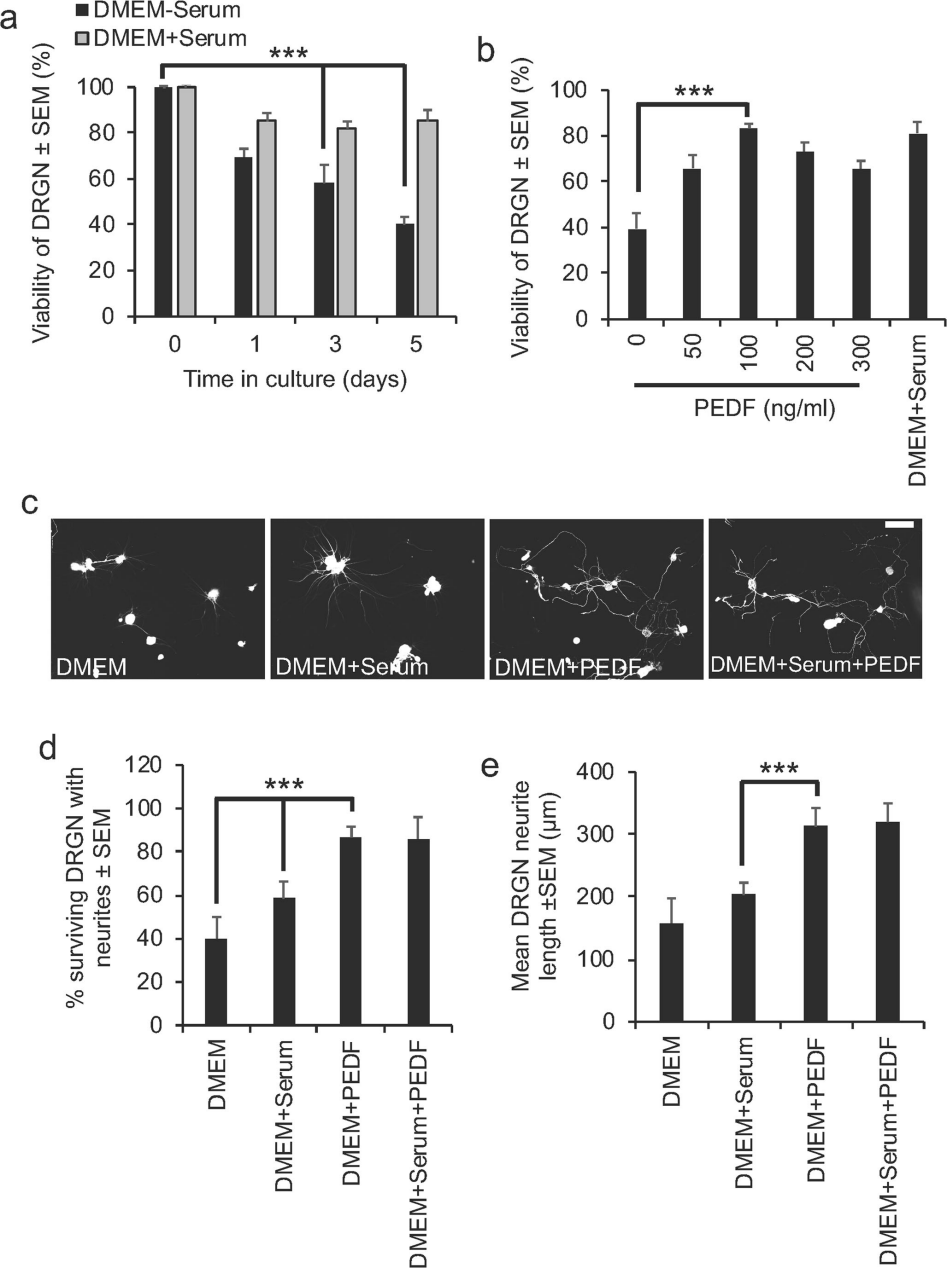
PEDF peptide promotes DRGN survival in a serum withdrawal model. **a** Approximately 40 and 60% of DRGN die after 3 and 5 days, respectively, when cultured in the absence of serum. **b** Supplementation with increasing concentrations of PEDF improved DRGN viability to 85% with 100 ng/ml PEDF after 5 days in culture, similar to that observed in the presence of serum. **c** Representative images to show DRGN neurite outgrowth after stimulation with 100 ng/ml PEDF, with and without serum. **d** DRGN survival and **e** mean neurite length after treatment with 100 ng/ml PEDF, with and without serum. Scale bar in **c** = 100μm. *** = *P* < 0.0001, ANOVA

To investigate the role of PEDF and its involvement in DRGN survival and neurite outgrowth, a serum-withdrawal DRGN culture model, in the presence of 5-FDU to keep glia involvement to a minimum, was used [5]. In this model, we first confirmed that 60% DRGN death occurred at 5 days after plating in DMEM without serum (Fig. 3a). DRGN viability at 5 days was then quantified in this serum-withdrawal model after DRGN culture with 0, 50, 100, 200, or 300 ng/ml PEDF peptide and tested for its ability to promote DRGN survival and neurite outgrowth. All doses of PEDF significantly enhanced DRGN viability up to a maximum observed with 100 ng/ml of PEDF, which promoted 83 ± 3% of DRGN survival (Fig. 3b). DRGN survival in the presence of 100 ng/ml PEDF caused > 50% more survival than that observed in the absence of PEDF (*P* < 0.0001) and was comparable to survival in the presence of serum (Fig. 3b). Concentrations of PEDF above 100 ng/ml reduced DRGN survival. These results suggest that PEDF rescues DRGN from death in a serum-withdrawal model.

### PEDF Is Neuritogenic for DRGN in a Serum Withdrawal Model

DRGN grown in the presence of 100 ng/ml of PEDF and 100μg/ml CME showed enhanced levels of disinhibited neurite outgrowth compared with those grown in DMEM and DMEM + Serum (Fig. 3c). However, there appeared to be no further enhancement in DRGN neurite outgrowth when grown in the presence of DMEM + Serum + PEDF (Fig. 3c). Quantification of the proportion of surviving DRGN with neurites showed that only 40 ± 10% and 58 ± 8% DRGN grew neurites when cultured in DMEM or DMEM + Serum, respectively (Fig. 3d). However, the proportion of DRGN with neurites increased significantly (*P* < 0.0001) when grown in the presence of either DMEM + PEDF reaching a maximum of 86 ± 10%, with no further additional enhancement in the proportion of DRGN growing neurites in the presence of DMEM + Serum + PEDF (Fig. 3d). The mean length of the longest neurites was also significantly greater when treated with DMEM + PEDF (312 ± 25 μm) compared with DMEM alone (158 ± 40 μm) or DMEM + serum (203 ± 20 μm) (*P* < 0.0001) (Fig. 3e). Treatment with DMEM + Serum + PEDF did not further potentiate neurite outgrowth when compared with DMEM + PEDF (Fig. 3e). These results demonstrate that 100 ng/ml PEDF promotes significant DRGN neurite outgrowth.

### AAV-Mediated Overexpression of PEDF Promotes DC Axon Regeneration and Functional Recovery

**Fig. 4:**
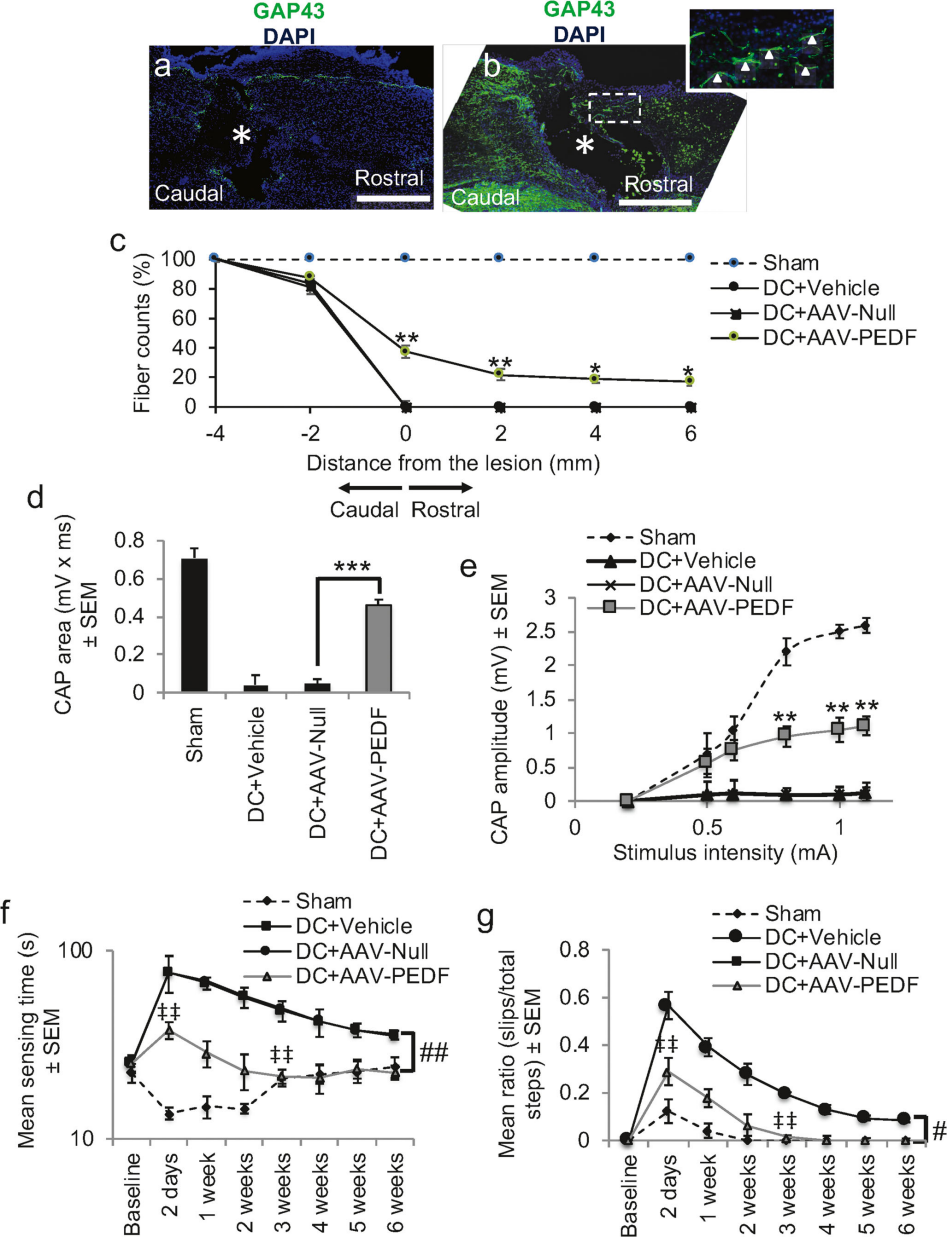
AAV8-mediated overexpression of PEDF promotes DC axon regeneration and improves functional recovery. **a** Few if any, GAP43^+^ fibres were present in the caudal or rostral segments of the cord (* = lesion site) in DC + AAV-Null-treated rats. **b** Numerous GAP43^+^ axons were present in the caudal and rostral segments of the cord after AAV-PEDF treatment. **c** Quantification of the total number of GAP43^+^ fibres at different distances caudal and rostral to the DC injury site. **d** CAP area and **e** CAP amplitudes were significantly improved after AAV-PEDF treatment. **f** Mean sensing time for the tape removal and **g** horizontal ladder walking tests both showed improvements in AAV-PEDF-treated rats and no significant differences in both tests by 3 weeks when compared with the Sham controls. Scale bars in **a** and **b** = 200 μm. * = *P* < 0.05, ** = *P* < 0.001, *** = *P* < 0.0001, ANOVA; ‡‡ = *P* < 0.001, (independent sample *t* test (DC + AAV-Null versus DC + AAV-PEDF at 2 days); # = *P* < 0.01, generalised linear mixed models; ## = *P* < 0.001, linear mixed models

Intra-DRG injection of AAV-PEDF significantly increased PEDF mRNA levels by 8.6 ± 0.5-fold compared with only 0.1 ± 0.05-fold mRNA in DC + AAV-Null-treated rats (Supplementary Fig. 1a). The levels of PEDF protein also increased to 687 ± 29 ng/mg tissue compared with 11 ± 7 ng/mg of tissue in the DC + AAV-Null-treated rats (Supplementary Fig. 1b). These results demonstrated that significant titres of PEDF mRNA and protein were induced in DRGN after intra-DRG injection of AAV-PEDF. GAP43 immunohistochemistry showed little or no GAP43^+^ regenerating fibres (green) in DC + AAV-Null-treated rats (Fig. 4a; inset shows high power of boxed region). However, after treatment with AAV-PEDF, significant numbers of GAP43^+^ regenerating axons were observed in the caudal and rostral segments of the spinal cord (Fig. 4b). Quantification of the number of GAP43^+^ fibres showed that AAV-PEDF promoted the regeneration of 38 ± 5, 22 ± 4, 19 ± 3, and 17 ± 3% of GAP43^+^ fibres at 0, 2, 4, and 6 mm rostral to the lesion site (Fig. 4c).

Electrophysiology showed that the CAP area observed in Sham (0.71 ± 0.2 mV × ms) reduced by 94% to 0.04 ± 0.05 mV × ms in DC + AAV-Null-treated rats (Fig. 4d). However, CAP area was significantly improved (*P* < 0.0001) in the AAV-PEDF-treated groups to 64% of that observed for the Sham controls (Fig. 4d). The mean CAP amplitude was also significantly reduced in the DC + AAV-Null-treated groups compared with the Sham controls (Fig. 4e). However, significantly larger CAP amplitudes were observed in DC + AAV-PEDF-treated rats at all stimulation intensities, compared with those in the DC + AAV-Null groups (*P* < 0.001; Fig. 4e).

The mean sensing time for the tape removal test was between 10 and 26 s in the Sham-treated controls throughout the 6-week time course (Fig. 4f). However, in the DC + AAV-Null-treated groups, sensing time increased significantly to 77 ± 19 s at 2 days after DC injury (Fig. 4f). Sensing time decreased over the 6-week period in DC + AAV-Null-treated rats and remained at 36 ± 3 s at 6 weeks after DC injury. In contrast, AAV-PEDF-treated rats showed significantly reduced sensing time at 2 days after DC, taking only 38 ± 4 s to detect the tape (*P* < 0.001, independent sample *t* test) and were not significantly different with the Sham-treated rats by 3 weeks after DC (Fig. 4f). Over the whole time course, there was a significant reduction in the time taken to sense the adhesive tape in the DC + AAV-PEDF-treated compared with the DC + AAV-Null-treated animals (linear mixed model, *P* < 0.001).

Over a 6-week time course, there was a significant increase in the error rates during the horizontal ladder walking (generalised linear mixed model, *P* < 0.0011) (Fig. 4g) in DC + AAV-Null-treated compared with DC + AAV-PEDF-treated animals. The mean error ratio was significantly lower in DC + AAV-PEDF compared with DC + AAV-Null-treated animals at 2 days after injury (*P* < 0.001, independent sample *t* test) and at 3 weeks after DC injury (*P* < 0.001, independent sample *t* test) by which time the error rates were similar to that of the Sham controls. In the DC + AAV-Null-treated groups, error remained for the full 6-week duration (Fig. 4g). Taken together, these results showed that AAV-PEDF promoted DC axon regeneration that led to improvements in electrophysiological and sensory and locomotor function.

### PEI-Mediated Overexpression of PEDF Promotes Similar Functional Recovery as AAV

**Fig. 5:**
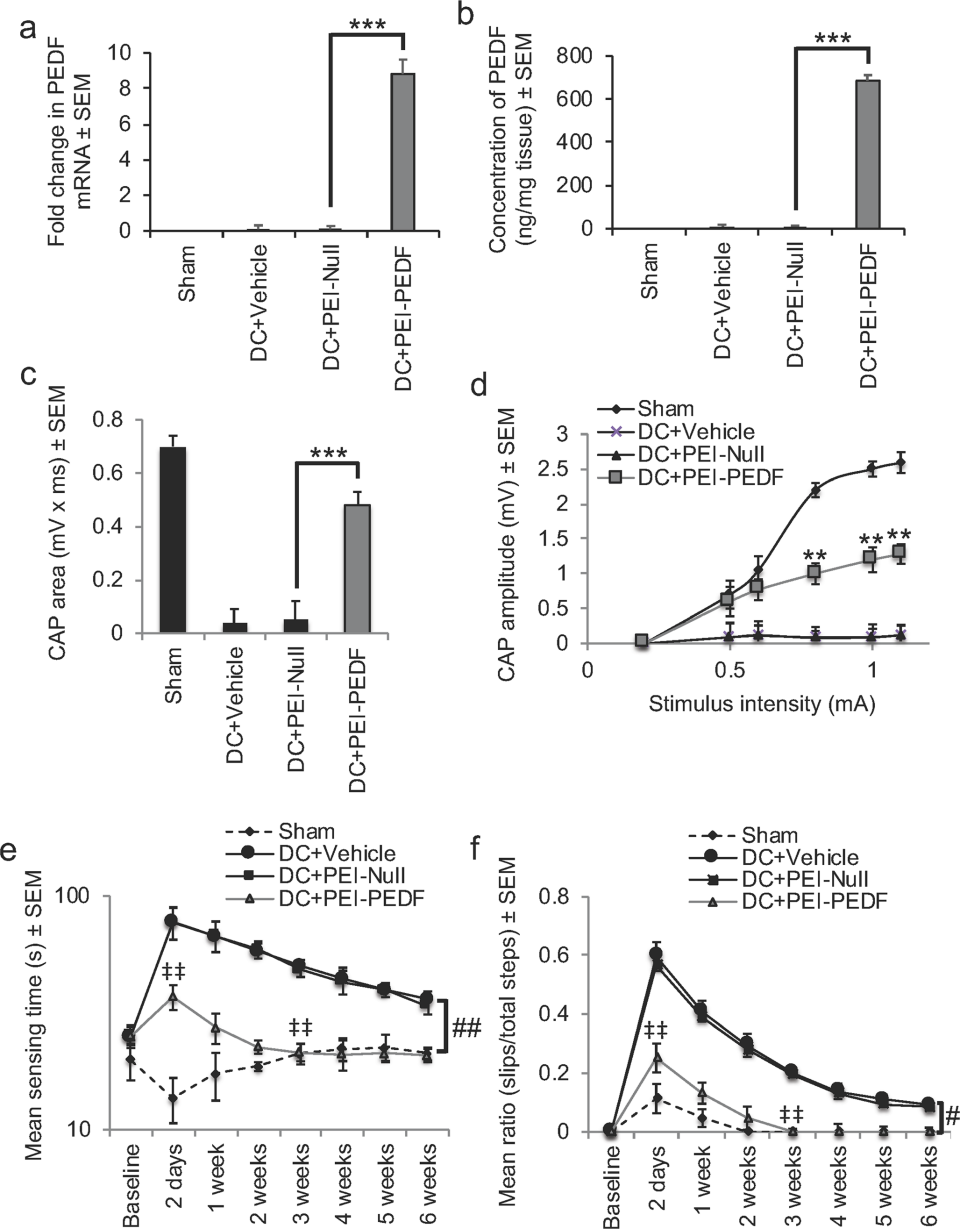
In vivo-jetPEI-mediated overexpression of PEDF improves similar levels of functional recovery after DC injury as AAV8. DC + PEI-PEDF treatment overexpresses PEDF **a** mRNA and **b** protein levels significantly compared with other treatment groups. **c** CAP area and **d** CAP amplitudes were significantly improved after DC + PEI-PEDF treatment. **e** Mean sensing time for the tape removal and **f** horizontal ladder walking tests both showed improvements in DC + PEI-PEDF-treated rats and no significant differences in both tests by 3 weeks when compared with the Sham controls. ** = *P* < 0.001, *** = *P* < 0.0001, ANOVA; ‡‡ = *P* < 0.001, independent sample *t* test (DC + PEI-Null versus DC + PEI-PEDF at 2 days); # = *P* < 0.01, generalised linear mixed models; ## = *P* < 0.001, linear mixed models

In the DC + PEI-PEDF groups, PEDF mRNA was significantly increased to 8.8 ± 0.8-fold (*P* < 0.0001) over that observed for either the Sham, DC + Vehicle, or DC + PEI-Null groups (Fig. 5a). Likewise, significant levels of PEDF protein were extracted from DC + PEI-PEDF-treated DRG, with 698 ± 26 ng/mg compared to only 11 ± 5 and 10 ± 4 ng/mg of tissue from DC + Vehicle and DC + PEI-Null-treated DRG, respectively (Fig. 5b). These results show that PEI-delivered plasmids overexpressed the expression of PEDF to similar levels in DRG as those observed with AAV8.

Electrophysiology also showed similar improvements in the CAP area (Fig. 5c) and the CAP amplitude (Fig. 5d) in the DC + PEI-PEDF-treated groups as those observed with AAV-PEDF (compare with Fig. 4d, e). For example, the CAP area in the DC + PEI-PEDF-treated groups improved by 69% of that observed for the Sham controls, whilst the mean CAP amplitudes were also significantly larger in the DC + PEI-PEDF-treated rats at all stimulation intensities, compared with those in the DC + PEI-Null groups (*P* < 0.001; Fig. 5d).

PEI-mediated overexpression of PEDF also promoted similar levels of sensory and locomotor functional recovery as AAV. For example, in the DC + PEI-PEDF-treated rats, the mean sensing time was 37 ± 6 s at 2 days after DC injury (*P* < 0.001, independent sample *t* test) and sensing times were not significantly different with the Sham-treated rats by 3 weeks after injury (Fig. 5e; *P* < 0.001, linear mixed model over the whole time course). The mean error ratios for the horizontal ladder crossing test were also significantly lower in the DC + PEI-PEDF-treated compared with the DC + PEI-Null-treated rats at 2 days after injury (*P* < 0.001, independent sample *t* test), and by 3 weeks after injury, the error rates were similar with that of the Sham controls (generalised linear mixed model, *P* < 0.001) (Fig. 5f). Taken together, these results demonstrate that PEI-mediated overexpression of PEDF was just as efficient as AAV in promoting improvements in electrophysiological, sensory, and locomotor function after DC injury in rats.

### Addition of Exogenous PEDF Enhances NTF in DRGN Cultures

**Fig. 6:**
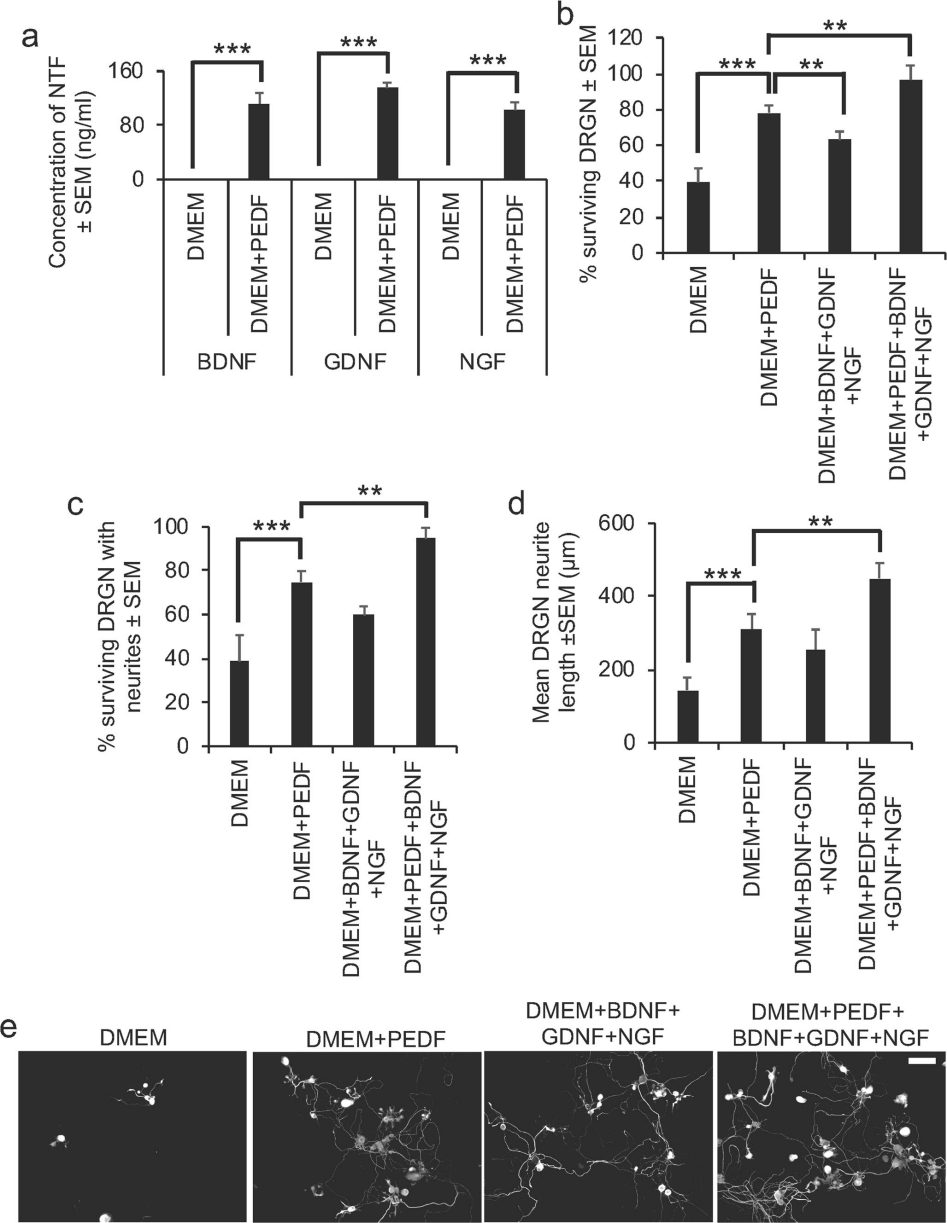
PEDF treatment promotes NTF secretion in DRGN cultures. **a** ELISA was used to detect high levels of BDNF, GDNF, and NGF after exposure of DRGN to 100 ng/ml PEDF in culture supernatant. **b** Exposure of DRGN cultures to ELISA-detected levels of BDNF + GDNF + NGF and PEDF + BDNF + GDNF + NGF all promotes similarly high levels of DRGN survival and **c** the number of DRGN with neurites. However, **d** mean neurite length is further potentiated by addition of PEDF compared with DMEM + PEDF-treated cultures. **e** Representative images to show DRGN neurite outgrowth after treatment with DMEM, DMEM + PEDF, DMEM + BDNF + GDNF + NGF, and DMEM + PEDF + BDNF + GDNF + NGF. Scale bar in **e** = 100 μm. ** = *P* < 0.001, *** = *P* < 0.0001, ANOVA

To expound a possible mechanism for the neuroprotective and axogenic properties of PEDF, we used dissociated DRGN cultures, grown in the presence of 5-FDU to limit glial proliferation and CME to mimic the post-injury environment of the degenerating spinal cord to investigate secretion of NTF into the culture medium. We determined that 100 ng/ml of PEDF caused the secretion of 112 ± 16, 135 ± 8, and 102 ± 12 ng/ml of BDNF, GDNF, and NGF (*P* < 0.0001 DMEM versus DMEM + PEDF) into the culture medium, respectively (Fig. 6a). No ciliary neurotrophic factor (CNTF) nor neurotrophin-3 (NT3) were detected in the culture medium (not shown). Reconstituting the isolated NTF in their relative concentrations in freshly prepared DRGN cultures, grown in the presence of CME, we observed significantly increased DRGN survival (by > 50%; Fig. 6b), number of surviving DRGN with neurites (i.e. neurite initiation—by 50%; Fig. 6c), and the mean neurite length (i.e. neurite elongation) from 143 ± 35 μm in DMEM alone to 312 ± 40 μm in DMEM + BDNF + GDNF + NGF-treated cultures (Fig. 6d). The levels of DRGN survival and neurite outgrowth were similar to that observed with DMEM + 100 ng/ml PEDF treatment (Fig. 6b–d). The addition of PEDF to DMEM + BDNF + GDNF + NGF-treated DRG cultures did not further potentiate DRGN survival (Fig. 6b) nor the number of DRGN with neurites (Fig. 6c), but significantly increased (*P* < 0.0001) mean neurite length to 449 ± 42 μm (*P* < 0.001), compared with DRGN neurite outgrowth in DMEM + PEDF or DMEM + BDNF + GDNF + NGF-treated cultures (Fig. 6d, e). These results suggest that PEDF-stimulated NTF, released from DRGN cultures, can promote significant DRGN neurite outgrowth and survival, similar to PEDF-alone-treated cultures, but that the addition of PEDF to BDNF + GDNF + NGF can further potentiate DRGN neurite length (elongation) but not survival nor the number of DRGN with neurites (initiation).

### Trk-Dependent Neurotrophins Are Responsible for some of the Neuroprotective and Neuritogenic Properties of PEDF

**Fig. 7:**
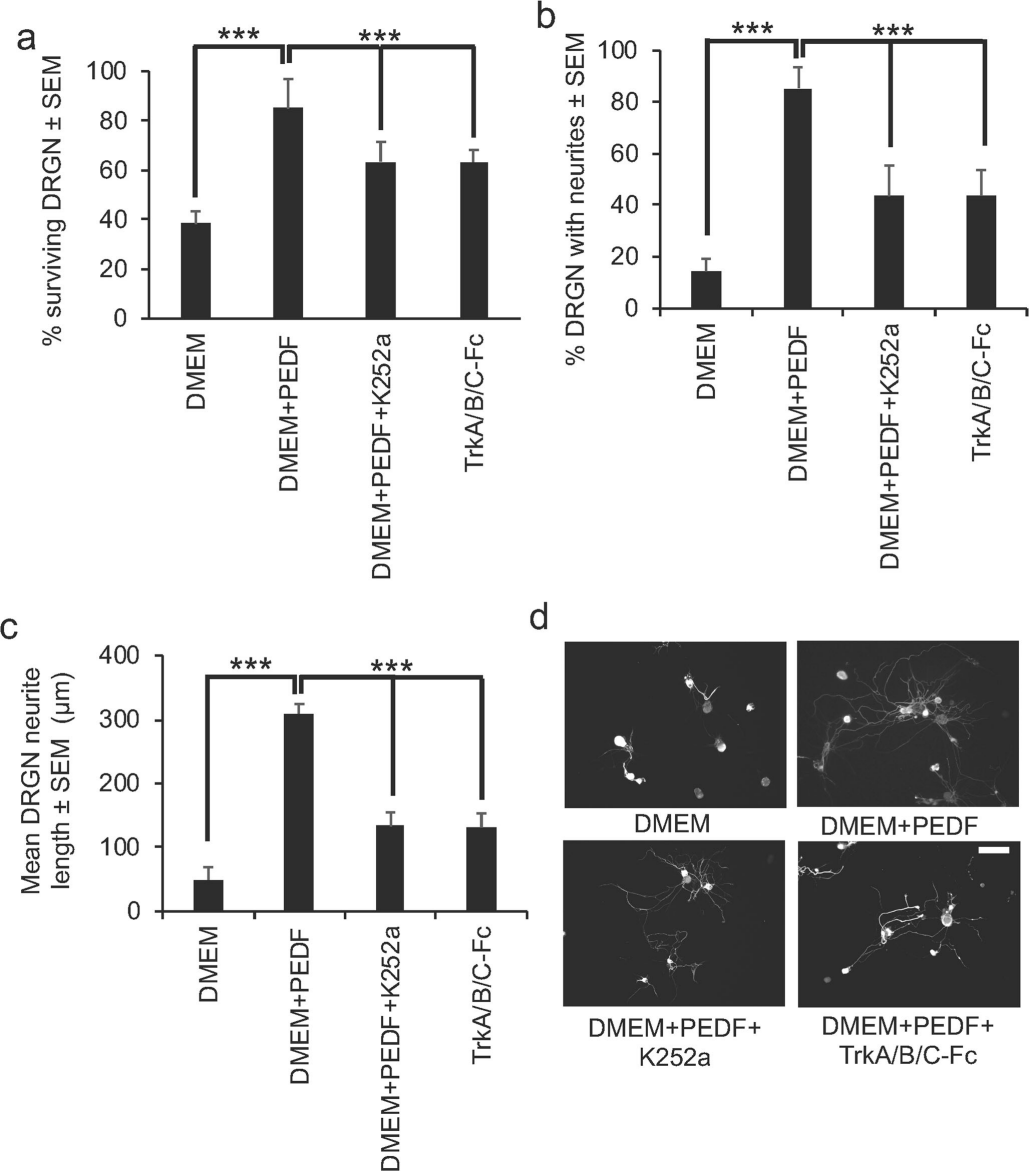
K252a and TrkA/B/C-Fc fusion proteins partially abrogate PEDF-stimulated DRGN survival and neurite outgrowth. K252a and TrkA/B/C-Fc fusion proteins significantly reduce **a** DRGN survival **b** % DRGN with neurites and the **c** mean neurite length. **d** Representative images to show partial suppression of DRGN neurite outgrowth after treatment with K252a and TrkA/B/C-Fc fusion proteins. Scale bar in **d** = 100 μm. *** = *P* < 0.0001, ANOVA

In dissociated DRGN cultures, grown in the presence of 5-FDU and CME, the neuroprotective effects of PEDF can be partially abrogated by either treatment with K252a, a broad-spectrum inhibitor of Trk-dependent neurotrophins or TrkA/B/C-Fc fusion proteins (specific inhibitors of TrkA/B/C [21]) (Fig. 7a). DRGN survival reduced from 85 ± 12 to 64 ± 5 and 63 ± 8% in DMEM + PEDF, DMEM + PEDF + K252a, and DMEM + TrkA/B/C-Fc fusion proteins, respectively. Likewise, K252a and TrkA/B/C-Fc fusion protein treatment only partially suppressed PEDF-stimulated DRGN neurite outgrowth in terms of the number of DRGN with neurites (Fig. 7b) and the mean neurite length (Fig. 7c, d). For example, the mean proportion of DRGN with neurites reduced from 83 ± 12 to 44 ± 8% and the mean DRGN neurite length reduced from 310 ± 15 to 133 ± 22 μm after treatment with K252a and TrkA/B/C-Fc fusion proteins. These results suggest that DRGN survival and neurite outgrowth are directly and indirectly affected by PEDF and that some of the effects are Trk-dependent.

## Discussion

Here, we have investigated PEDF as a novel target for promoting DRGN survival and neurite outgrowth/axon regeneration and functional recovery in the injured spinal cord. We utilised in vivo regenerating and non-regenerating DRGN models and the in vitro serum-withdrawal model of DRGN cultures, to investigate how the expression of PEDF is modulated in DRGN with contrasting intrinsic growth capacities. We showed that (1) PEDF expression in DRGN in vivo is upregulated in regenerating (SN, pSN + DC) models; (2) PEDF-R is expressed in DRGN in vivo; (3) PEDF promotes significant DRGN survival and disinhibited DRGN neurite outgrowth in a serum-withdrawal model and in the presence of inhibitory CME; (4) intra-DRG delivery of AAV-PEDF promoted significant DC axon regeneration and electrophysiological, sensory, and locomotor function; (5) intra-DRG delivery of PEI-PEDF (non-viral vector) promoted similar electrophysiological and functional recovery as AAV8 (viral vector delivery); (6) exposure of DRGN to PEDF in vitro causes the release of significant titres of BDNF, GDNF, and NGF and that these concentrations of NTF significantly enhanced disinhibited DRGN neurite outgrowth; and (7) PEDF-induced DRGN survival and neurite outgrowth are partially suppressed by Trk receptor blockers.

To our knowledge, this is the first study in sensory DRGN to demonstrate a correlation between endogenous upregulation of PEDF and survival/axonal regeneration in DRGN and the ascending DC pathway. PEDF was previously shown to be endogenously upregulated in activated Müller cells and contributed to retinal ganglion cell (RGC) survival in several vision-threatening pathologies [24]. We also showed that PEDF was RGC-neuroprotective and promoted significant RGC axon regeneration in vivo and that PEDF protein was present in RGC, astrocytes and Muller cells in the retina [5, 6]. In contrast, this study has observed that endogenous expression of PEDF was restricted to DRGN and was absent in satellite cells. This study demonstrated both intracellular and extracellular activity for PEDF in regenerating DRGN paradigms.

Results from immunohistochemistry did not identify the presence of an extracellular PEDF-R in DRG satellite cells; PEDF-R^+^ immunostaining was only present around the cell membranes of DRGN. However, the PEDF-R has not been fully characterised, and it is possible that PEDF acts at an alternative, as yet unidentified receptor on satellite cells, which is not detected by the antibody used in this and other previous studies [25, 26]. PEDF itself, however, was first identified as an NTF secreted by retinal pigment epithelial cells and was originally regarded as an extracellular signalling protein [27]. Concurrent with more recent studies, we detected intracellular PEDF with transient PEDF^+^ nuclear immunostaining [28, 29]; the nuclear translocation motif of PEDF is mediated by transportin-SR2 [30]. Such an uptake mechanism may be important in mediating the neuroprotective effects of PEDF through further interactions within the nucleus. This would explain our observations that nuclear expression of PEDF is only present in regenerating (SN, pSN + DC) models and not in a non-regenerating (DC) model or in the intact control DRGN.

Our study also demonstrated that exogenous PEDF promoted DRGN survival and disinhibited DRGN neurite outgrowth in a serum-withdrawal in vitro model and is the first to show these effects in spinal neurons of adult rats. In addition, AAV-mediated delivery of PEDF to injured DRGN-enhanced DC axon regeneration and improved electrophysical, sensory, and locomotor function in treated rats. Moreover, non-viral vector-mediated plasmid delivery through the use of in vivo-jetPEI immediately after injury also promoted similar electrophysiological, sensory, and locomotor functional recovery as AAV8-mediated overexpression of PEDF. These results are in accordance with our previous studies in other populations of neurons, where PEDF was neuroprotective and axogenic in RGC both in vitro and in vivo [5, 6].

Through PEDF pathway analysis, we identified an upregulation of NFκB mRNA concurrent with the upregulation of PEDF in regenerating pSN + DC DRGN. Notably, multiple NTF and the anti-apoptotic gene Bcl-2 were also upregulated in this model. As a transcription factor with a nuclear colocalisation signal, activated NFκB mediates upregulation of NTF and anti-apoptotic genes/proteins [31]. NFκB activation is a critical step in preventing neuronal death via a number of pathways throughout the CNS, most notably by mediating the neuroprotective effects of PEDF in cerebellar granule neurons [32]. It is therefore possible that NFκB is responsible for mediating some of the neuroprotective and axogenic effects of PEDF in sensory neurons, through the upregulation of NTF and anti-apoptotic genes/proteins. In this regard, BDNF, GDNF, and NGF were secreted from DRGN in response to PEDF and blockade of Trk receptors, and hence, these NTF do not account for all of the survival and neurite outgrowth-promoting effects of PEDF and suggests that PEDF has both direct and indirect effects on DRGN.

Viral vectors, such as AAV8, are commonly used to deliver genes into the CNS but are restricted by insert capacity and 7–14 days delay in optimal target mRNA expression, limiting their therapeutic effect and their translational potential for use in acute conditions [33–37]. However, non-viral vectors such as in vivo-jetPEI are able to transfect cells in vitro and in vivo, are easy to prepare, stable, and safe; and give immediate therapeutic benefits [38–40]. For example, melanopsin knockdown using in vivo-jetPEI-delivered shRNA was observed within 16 h after transduction in the eye and lasted for at least 2 months [41]. We used in vivo-jetPEI and monitored *gfp* expression in DRGN after DC injury and found that in vivo-jetPEI transduced similar proportions of large diameter DRGN as AAV8, without invoking a non-specific innate immune response [15, 16]. Given the advantages of in vivo jetPEI over viral vectors, PEDF overexpression using such a non-viral vector presents itself as an exciting therapeutic opportunity to improve functional recovery in spinal cord injury affected patients.

In conclusion, this is the first study to demonstrate that PEDF is an important mediator of DC axon regeneration in the adult mammalian system. We have demonstrated that PEDF is neuroprotective and promotes significant DRGN neurite outgrowth, exhibiting both direct and indirect effects on DRGN. As such, PEDF shows promise to be a potentially novel therapy for neuroprotection and axogenesis after SCI.

## Electronic supplementary material

Supplementary Figure 1 AAV-PEDF stimulates production of PEDF in DRG. (**a**) AAV-PEDF significantly overexpresses PEDF mRNA and (**b**) protein when compared to DC+AAV-Null-treated rats and leads to production of 50% more PEDF when compared to pSN+DC-treated rats. (PNG 28 kb)

High resolution image (TIFF 170 kb)
