# Supplementary figures and images for: Pigment Epithelium-Derived Factor Promotes Axon Regeneration and Functional Recovery After Spinal Cord Injury
Source: Mol Neurobiol. 2019 May 2;56(11):7490–507. doi: 10.1007/s12035-019-1614-2 (PMC6815285; doi:10.1007/s12035-019-1614-2)

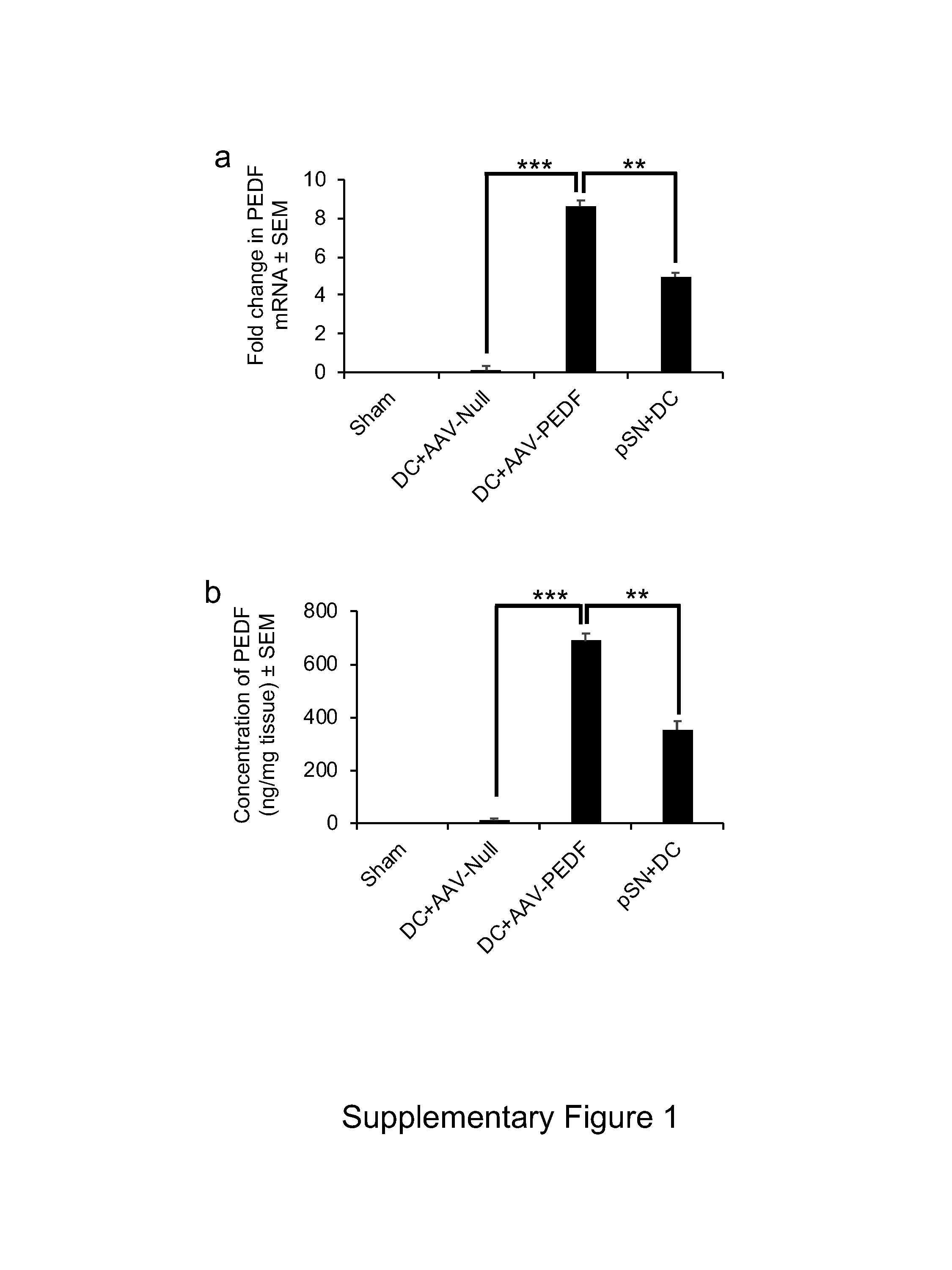

Supplement: Supplementary file 1 — AAV-PEDF stimulates production of PEDF in DRG. (a) AAV-PEDF significantly overexpresses PEDF mRNA and (b) protein when compared to DC+AAV-Null-treated rats and leads to production of 50% more PEDF when compared to pSN+DC-treated rats. (PNG 28 kb) [file 12035_2019_1614_Fig8_ESM.png]

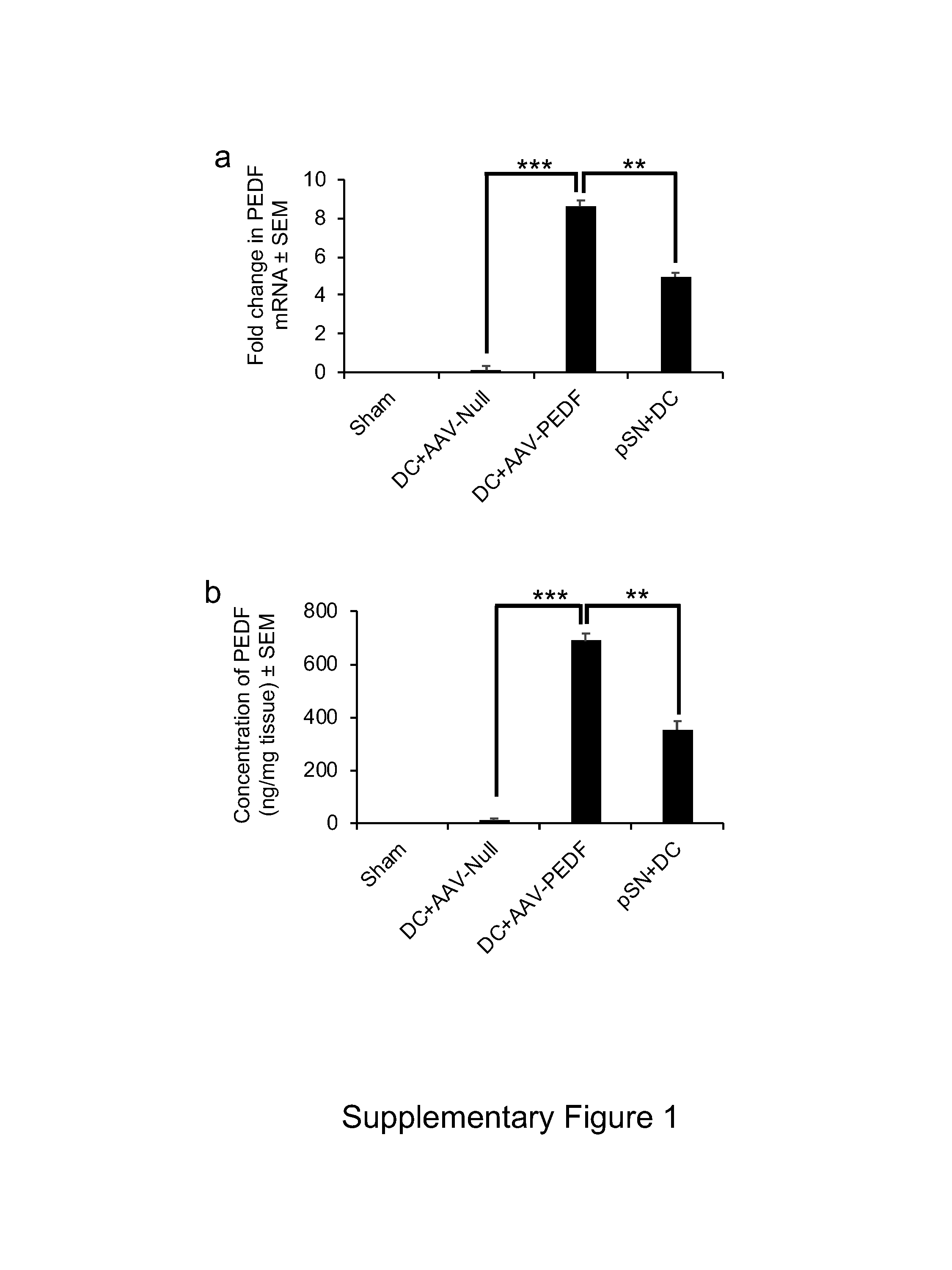

Supplement: Supplementary file 2 — (TIFF 170 kb) [file 12035_2019_1614_MOESM1_ESM.tiff]
